# Myelin oligodendrocyte glycoprotein antibody-associated disease presenting as encephalitis and concurrent Epstein-Barr virus detection: a case series

**DOI:** 10.3389/fimmu.2026.1788238

**Published:** 2026-05-08

**Authors:** Runzhe Chen, Xianli Song, Yupeng Du, Sijia Hou, Minzhi Zhang, Rui Liu, Huihui Xue, Jinming Li, Xiaona Xu, Ting Li, Bohao Zhang, Chun-Sheng Yang, Wei Jiang

**Affiliations:** 1 Department of Neurology, Tianjin Medical University General Hospital, Tianjin, China; 2 Department of Medicine Research Center, The Third Affiliated Hospital of Zhengzhou University, Zhengzhou, Henan, China; 3 Department of Radiology, The Third Affiliated Hospital of Zhengzhou University, Zhengzhou, Henan, China

**Keywords:** demyelinating diseases, encephalitis, Epstein-Barr virus, MOG-IgG, myelin oligodendrocyte glycoprotein antibody-associated disease

## Abstract

Myelin oligodendrocyte glycoprotein (MOG) antibody-associated disease (MOGAD) is an autoimmune disorder affecting the central nervous system (CNS), characterized by the presence of autoantibodies against MOG (MOG-IgG) in serum or cerebrospinal fluid (CSF). While Epstein-Barr virus (EBV) is implicated in multiple sclerosis (MS), its role in MOGAD remains unclear. Here, we report five cases of newly diagnosed MOGAD manifesting as encephalitis, in whom concurrent EBV-related laboratory findings were detected. All patients tested positive for serum MOG-IgG by live cell-based assay (CBA). Regarding therapeutic response, three patients exhibited transient improvement in CSF parameters following initial antiviral therapy. However, clinical recovery was observed only after administration of corticosteroids (either high- or low-dose). In refractory cases, additional interventions with intravenous immunoglobulin (IVIG) or tocilizumab were applied. In conclusion, this case series suggests that EBV-associated immune activation may coexist with some MOGAD patients presenting as encephalitis, which requires validation in larger prospective and mechanistic studies in the future.

## Introduction

Myelin oligodendrocyte glycoprotein (MOG) antibody-associated disease (MOGAD) is a rare antibody-mediated inflammatory demyelinating disorder of the central nervous system (CNS) (1). At present, the diagnosis of MOGAD relies on the identification of core clinical demyelinating events, a positive MOG-IgG test, corroborative clinical or magnetic resonance imaging (MRI) findings, and the exclusion of other demyelinating disorders such as multiple sclerosis (MS) (2). Despite these diagnostic criteria, the etiology and underlying pathophysiological mechanisms of MOGAD remain poorly understood. Epstein–Barr virus (EBV), a herpesvirus mainly infecting B lymphocytes, is associated with various malignancies as well as autoimmune diseases. Notably, while a causal relationship has been established between EBV infection and MS (3, 4), the evidence indicating links between EBV and MOGAD is insufficient, and findings from MS cannot be simply extrapolated to MOGAD due to their distinct clinical and immunological features (1, 2). Recently, we identified five patients with MOGAD who initially presented with encephalitis and exhibited concurrent EBV-related laboratory evidence. Notably, these patients demonstrated late seroconversion or dynamic changes in MOG-IgG titers during their clinical course. This observation provides a clue for the potential association between EBV and MOGAD, which deserves further prospective and mechanistic studies.

## Case description

### Patient 1

**Figure 1:**
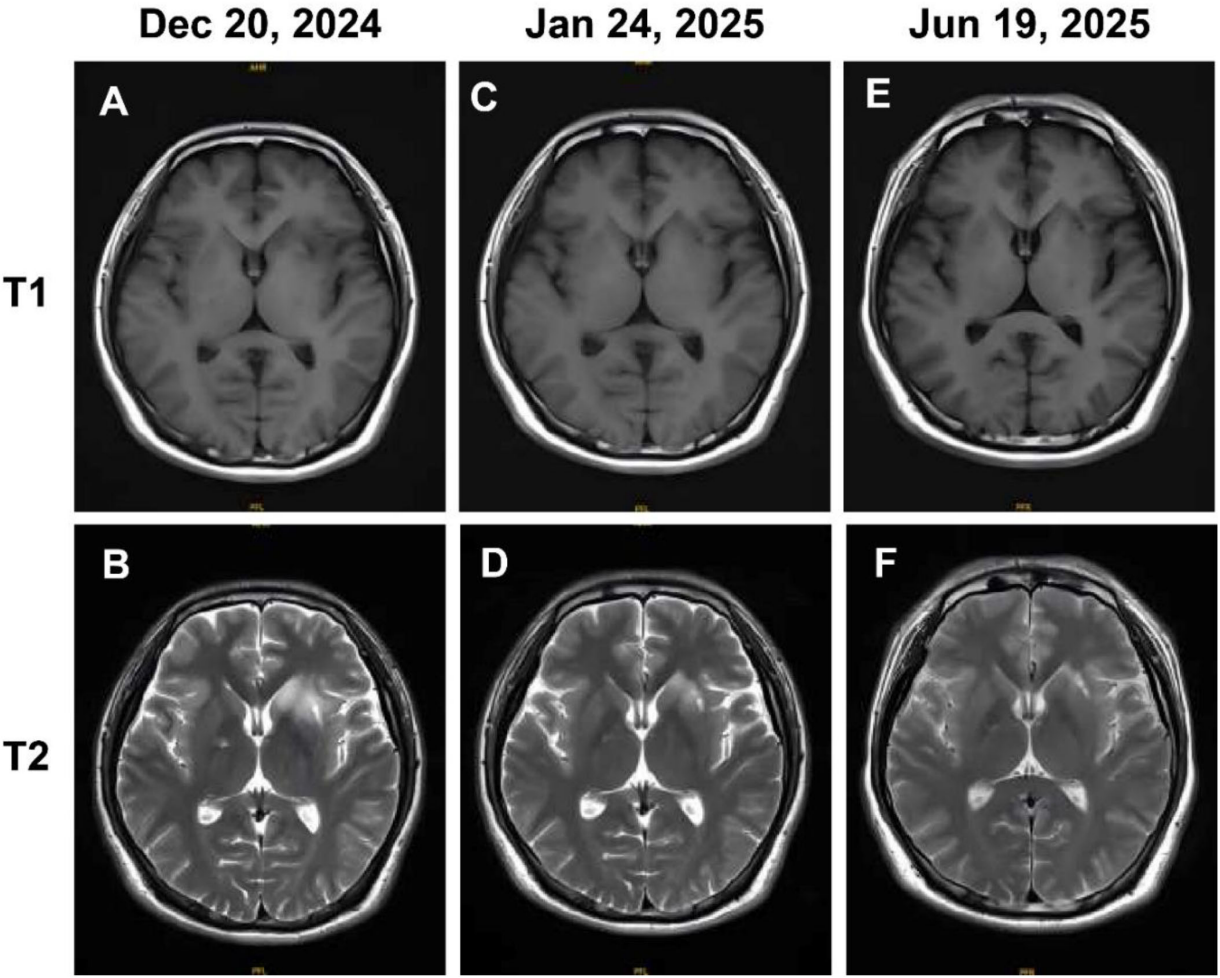
Brain MRI images of patient 1. **(A, B)** Images obtained prior to intravenous methylprednisolone and tocilizumab therapy. **(C, D)** Follow-up MRI scans conducted a month post-treatment. **(E, F)** Follow-up images acquired six months post-treatment with regular tocilizumab infusions.

A 29-year-old male presented with a primary complaint of fever and headache lasting over five days. Lumbar puncture showed CSF with elevated protein (0.95 g/L) and markedly increased white blood cell (WBC) count (477 cells/mm³). Serological testing showed that the patient was positive for Epstein-Barr nuclear antigen (EBNA)-IgG and negative for viral capsid antigen (VCA)-IgM. Metagenomic next-generation sequencing (mNGS) of CSF confirmed the presence of EBV sequences, with 25 reads detected. Testing for MOG-IgG was positive in both serum and CSF, with titers of 1:10 in each, while other autoimmune encephalitis antibodies were negative. Contrast-enhanced brain MRI revealed abnormal signal intensities in the bilateral basal ganglia, frontal lobes, and left insular cortex. However, the patient experienced symptomatic exacerbation despite initial intravenous immunoglobulin (IVIG) therapy (30 g/day for five days). Serum MOG-IgG increased to 1:100, and MRI scans continued to show abnormal signals (**Figures 1A, B**). Based on these findings, a diagnosis of MOGAD was established. The patient was subsequently treated with intravenous methylprednisolone (80 mg daily for one week) combined with tocilizumab (8 mg/kg), and clinical improvement was observed thereafter. A follow-up MRI performed one month later demonstrated a significant reduction in lesion burden (**Figures 1C, D**). Three months after discharge, serum MOG-IgG became negative. Further imaging at six months post-treatment revealed continued lesion improvement with regular tocilizumab infusions and a gradual steroid taper (**Figures 1E, F**). The patient has remained relapse-free to date.

### Patient 2

**Figure 2:**
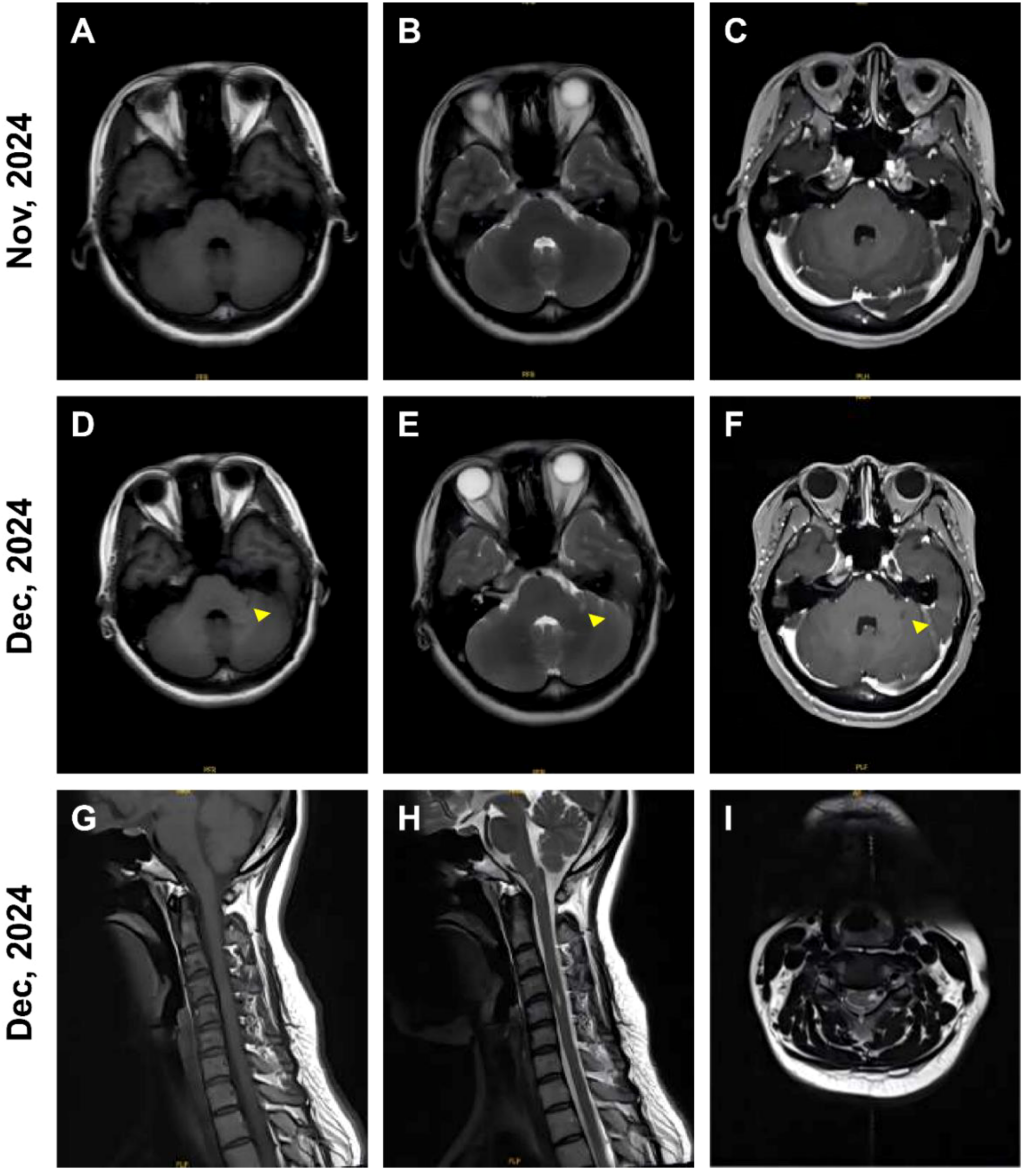
Imaging features of patient 2. **(A-C)** At the initial admission, brain MRI showed no notable abnormalities. **(D-F)** At the second admission, brain MRI revealed new lesions in the left cerebellar hemisphere and left middle cerebellar peduncle. **(G-I)** Cervical spinal cord MRI displayed abnormal signal intensities in the lower medulla oblongata and at the C4-C5 levels.

A 32-year-old female presented with a three-week history of fever, headache and sore throat. Serological testing at a primary hospital was positive for VCA-IgM, VCA-IgG, and EBNA-IgG. Physical examination on admission revealed negative meningeal signs. CSF analysis showed leukocytosis (72 cells/mm³) and increased protein (0.36 g/L), whereas mNGS of EBV, bacterial culture, Gram staining, and AFB staining were all negative in the CSF. Brain MRI demonstrated no significant abnormalities (**Figures 2A-C**). The patient was treated with penciclovir and symptomatic therapy. Prior to discharge, her clinical symptoms had improved. However, physical examination identified a localized cervical lymphadenopathy measuring 1.5 × 1.5 cm, characterized by firm consistency and absence of tenderness.

Two weeks post-discharge, the patient developed new-onset bilateral blurred vision accompanied by numbness in the left limb. These symptoms persisted and worsened despite supportive care. Neurological examination revealed a positive relative afferent pupillary defect (RAPD) sign in the right eye, mild left lower limb ataxia, and resolution of the previously observed cervical lymphadenopathy. Repeat CSF analysis showed mild leukocytosis (10 cells/mm³) and further elevations of protein levels (0.41 g/L). Visual evoked potentials (VEP) indicated optic nerve impairment, more pronounced on the right side. Brain MRI identified new abnormal signals in the left cerebellar hemisphere and peduncle (**Figures 2D-F**). Cervical spinal cord MRI revealed abnormal signal intensities in the lower medulla oblongata and at the C4-C5 levels (**Figures 2G-I**). MOG-IgG testing was positive in both serum (1:320) and CSF (1:10), while OCB and other autoimmune encephalitis antibodies were negative. Retrospective analysis of the CSF sample obtained during the prior hospitalization also confirmed MOG-IgG positivity (serum 1:100, CSF 1:1). Given the typical presentations of MOGAD, the patient received high-dose corticosteroid pulse therapy followed by maintenance treatment with tocilizumab (8 mg/kg). After discharge, she received regular monthly infusions of tocilizumab at 8 mg/kg. Her serum MOG-IgG titer decreased to 1:10 after six months and became seronegative at one year. She has remained clinically relapse-free during follow-up to date.

### Patient 3

A 38-year-old male presented with three separate episodes of recurrent encephalitis occurring over a span of two years. The initial episode, occurring between March and April 2023, was marked by acute-onset fever, bilateral temporal headache, vomiting, and agitation. Physical examination revealed negative meningeal signs. The CSF was notable for lymphocytic-dominant pleocytosis (300 cells/mm³, 85% lymphocytes), elevated protein concentration (0.87 g/L) along with raised opening pressure (204 mm H_2_O). Bacterial culture, Gram staining, AFB staining, as well as NGS results were all negative. Contrast-enhanced brain MRI revealed no definitive abnormal enhancement within the brain parenchyma. However, T2-weighted fluid-attenuated inversion recovery (FLAIR) sequences showed mildly increased signal intensity in the bilateral occipital cortex. Both serum and CSF testing for OCB, MOG-IgG and other autoimmune encephalitis antibodies yielded negative results. The patient was diagnosed with encephalitis of unknown cause and demonstrated clinical improvement following empiric antiviral therapy.

During the second episode in June 2023, the patient experienced a recurrence of fever and headache. MRI findings were unremarkable, whereas CSF analysis revealed lymphocytosis (56 cells/mm³) and elevated protein levels (0.74 g/L). Symptomatic improvement was observed after administration of ganciclovir.

The third episode, which took place in November 2024, was preceded by an attack of febrile sinusitis and was characterized by recurring headaches and continuous low-grade fever. CSF analysis showed leukocytosis (300 cells/mm³) and elevated protein (0.57 g/L). Notably, serum MOG-IgG was positive at a titer of 1:32, while CSF MOG-IgG was negative. No other antibodies associated with autoimmune encephalitis or OCB were identified. Additionally, mNGS detected EBV DNA with 32 reads in serum and 2 reads in CSF. Repeat brain MRI demonstrated no significant changes compared to prior imaging. Based on these findings, a diagnosis of MOGAD was established. His symptoms subsided following initiation of low-dose corticosteroid therapy. After discharge, the patient underwent gradual corticosteroid tapering, and his serum MOG-IgG turned negative at 3 months. He has been relapse-free during the one-year follow-up to date.

### Patient 4

A 24-year-old male presented with a one-week history of worsening headache and fever that did not improve with initial symptomatic treatment. Brain MRI showed no clear abnormalities. CSF analysis demonstrated leukocytosis (58 cells/mm³), increased protein level (0.35 g/L), and elevated pressure (260 mm H_2_O).

CSF mNGS, bacterial culture, Gram staining, and AFB staining were all negative. Antiviral and intracranial pressure-lowering treatments were ineffective, and the symptoms continued to worsen. A follow-up CSF test showed elevated opening pressure (330 mm H_2_O) and WBC count (200 cells/mm³). These parameters, along with the clinical condition, subsequently improved following intravenous immunoglobulin (IVIG) therapy, after which the patient was discharged.

However, three weeks later, the patient’s headache exacerbated, accompanied by a seizure. T2-weighted MRI scans demonstrated hyperintense signals distributed along the frontal and parietal sulci. CSF analysis revealed an increased opening pressure (326 mm H_2_O), and pleocytosis (27 cells/mm³). Testing for MOG-IgG was positive in both serum (1:320) and CSF (1:10). mNGS detected a single unique read aligning to EBV in the CSF, while repeat bacterial, mycobacterial, OCB and other autoimmune antibody testing remained negative. A retrospective analysis of samples collected from initial hospitalization also confirmed the presence of MOG-IgG in both serum (1:100) and CSF (1:1). Based on these findings, a final diagnosis of MOGAD was established. The patient was subsequently treated with high-dose corticosteroids, leading to gradual clinical improvement. Following discharge, he received a gradual corticosteroid tapering regimen. His serum MOG-IgG titer decreased to 1:10 at 3 months and became negative at 6 months. He has remained clinically relapse-free during the one-year follow-up to date.

### Patient 5

**Figure 3:**
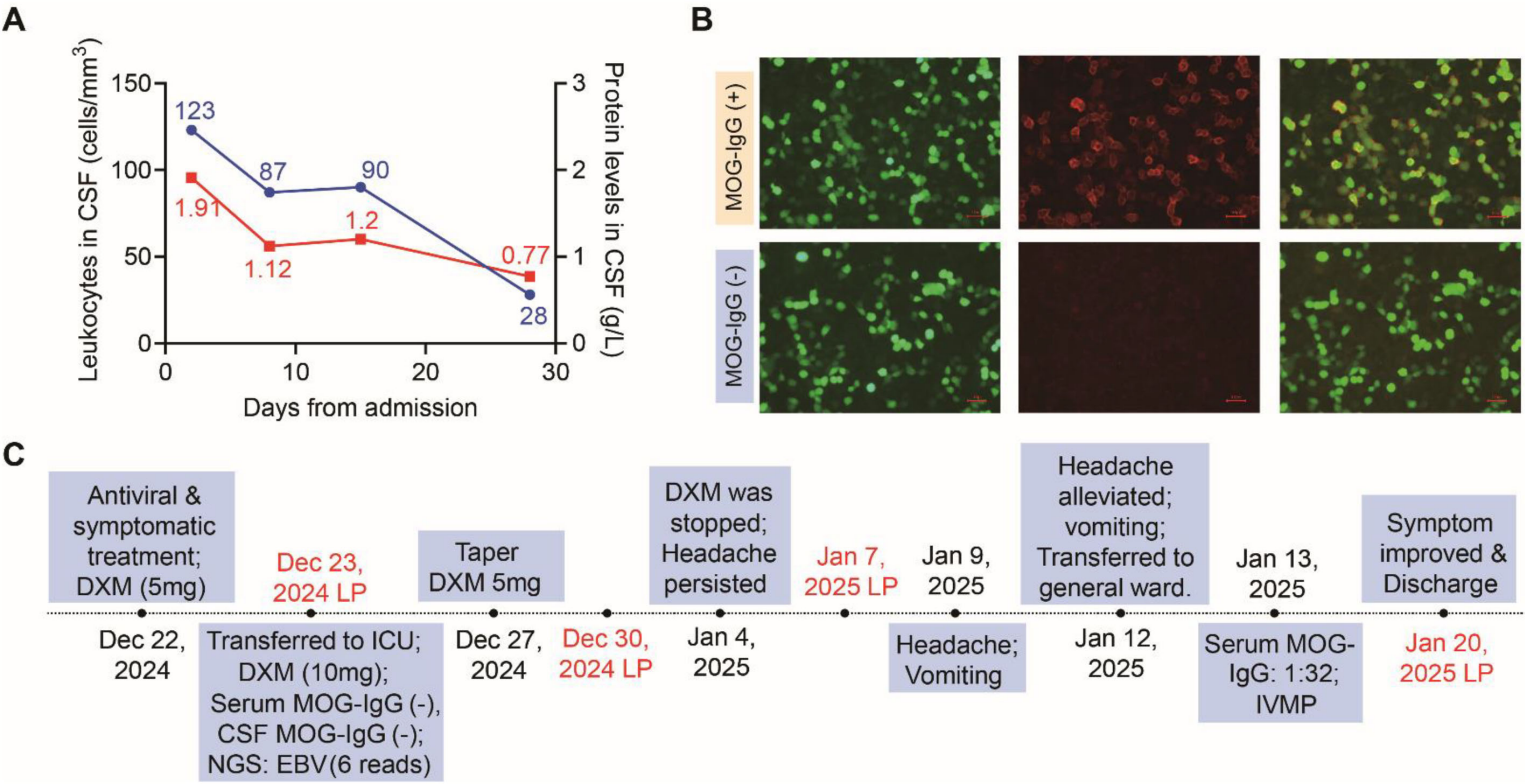
Clinical features of patient 5. **(A)** A line graph illustrating the dynamic changes in CSF leukocyte counts (blue) and protein concentrations (red) during the clinical course of Patient 5. **(B)** Serum MOG-IgG was positive at a titer of 1:32 by live CBA in January 2025, but negative in December 2024. Scale bar, 100μm. **(C)** Clinical course of Patient 5. Abbreviations: DXM, dexamethasone; LP, lumbar puncture; ICP, intracranial pressure; IVMP, intravenous methylprednisolone.

A 46-year-old male patient presented with a seven-day history of fever, headache, and experienced a seizure before admission. Physical examination revealed negative neurological signs. Despite empirical administration of sedation, antiepileptic, and antibiotic therapies, he continued to exhibit episodes of incoherent speech and agitation. Brain MRI demonstrated mild ventricular system dilation. Serologic testing revealed positive EBNA-IgG and VCA-IgG, with negative VCA-IgM. Initial CSF analysis indicated lymphocytic-predominant pleocytosis (WBC 123 cells/mm³), markedly elevated pressure (400 mm H_2_O) and protein level (1.91 g/L) (**Figure 3A**). Bacterial culture, Gram staining, and AFB staining were all negative. mNGS identified six unique EBV DNA reads in the CSF. Subsequent testing for OCB, autoimmune encephalitis antibodies, including MOG-IgG, yielded negative results in both serum and CSF. Due to ongoing headache and refractory vomiting, further CSF evaluations demonstrated persistently elevated pressure (400 mm H_2_O). Repeat MOG-IgG testing three weeks later was positive in serum (1:32) but still negative in CSF (**Figure 3B**), raising suspicion for MOGAD. Administration of low-dose methylprednisolone (80 mg daily for one week) resulted in significant clinical improvement, with cessation of seizure activity. Follow-up CSF analysis revealed decreases in cell count (28 cells/mm³) and protein level (0.77 g/L). The patient ultimately achieved full recovery, regaining normal communicative abilities and independent ambulation (**Figure 3C**). After discharge, the patient underwent gradual corticosteroid tapering, and his serum MOG-IgG became negative at 6 months. He has remained clinically relapse-free during follow-up to date.

## Discussion

This study describes five adults (aged 24–46 years; four males, one female) with MOGAD who initially presented with encephalitis-like manifestations, including fever and headache (**Table 1**). The initial diagnosis of encephalitis was based on the presence of fever and headache, along with elevated white blood cell count and protein levels in the cerebrospinal fluid (CSF), increased opening pressure, and positive EBV DNA in the CSF detected by metagenomic next-generation sequencing (mNGS), except for Patient 2 (who tested positive for VCA-IgM in serological assays) (5). However, a common feature among these cases was the occurrence of a second episode of MOGAD, with several patients exhibiting late seroconversion and dynamic rises in antibody titers over time–findings that, combined with characteristic MRI features and clinical manifestations, ultimately led to the MOGAD diagnosis (2). Of course, MOGAD can also present as encephalitis (1, 2). Although MOG-IgG positivity is a distinguishing feature, both the MOG-IgG titer and the associated clinical manifestations should be carefully interpreted, and close clinical follow-up is strongly recommended. Simultaneously, our findings suggest that MOG-IgG testing should be considered not only in patients with typical optic neuritis or myelitis, but also in those with unexplained encephalitis, particularly when EBV is detected by CSF mNGS – even at low read counts. In addition to symptomatic and empirical antiviral therapy, early initiation of immunomodulatory therapy may be crucial to reduce the risk of relapse in this clinical scenario.

**Table 1:** Summary of manifestations of the five patients.

Characteristic	Patient 1	Patient 2	Patient 3	Patient 4	Patient 5
Age, y/Sex	29/Male	32/Female	38/Male	24/Male	46/Male
Episodes No.	1	2	3	2	1
Clinical manifestations	Fever, headache	First: Fever, headache; Second: blurred vision and left limb numbness	Fever, headache, vomiting	First: fever, headache; Second: headache, seizure	Fever, headache, seizure
MRI findings	T2-FLAIR hyperintensity with mild enhancement in the bilateral basal ganglia, temporo-insular regions, and left cerebral peduncle	Punctate T2-hyperintensity in the left cerebellar and peduncle, with short-segment T2 hyperintensity in the C4-C5 of the spinal cord.	T2-FLAIR hyperintensity in the bilateral occipital cortex	T2-weighted hyperintensity along the frontal and parietal sulci	Mild ventricular system dilation
CSF parameters					
WBC/mm^3 ^a^^	477	72	300	58	123
Protein, g/L^a^	0.95	0.36	0.57	0.35	1.91
Glucose, mmol/L^a^	3.4	2.5	2.7	3.5	2.47
Chloride, mmol/L^a^	123	120	124	122	108
EBV evidence					
EBV antibody	VCA-IgM (-) EBNA-IgG (+)	VCA-IgM (+) VCA-IgG (+) EBNA-IgG (+)	NA	NA	VCA-IgM (-) VCA-IgG (+) EBNA-IgG (+)
EBV DNA reads ^b^	25 in CSF	Neg	First: Neg; Third: 32 in serum and 2 in CSF	First: Neg Second: 1 in CSF	6 in CSF
Time from symptom onset to EBV NGS detection in CSF(d)	10	24	Third: 15	Second: 12	8
MOG-IgG evidence					
MOG-IgG titer, serum/CSF	10 ^a^/10	First: 100/1 ^d^; Second: 320/10	Third: 32/Neg	First: 100/1 ^d^; Second: 320/10	32/Neg
Time from symptom onset to MOG-IgG detection (d)	10	Second: 5	Third: 15	Second: 12	27
OCB	Neg	Neg	Neg	Neg	Neg
Co-existing Ab	No	No	No	No	No
Autoimmune disease history	No	No	No	No	Psoriasis
Vaccine history ^e^	As scheduled	As scheduled	As scheduled	As scheduled	As scheduled
Smoking/Drinking	No/No	No/No	No/No	No/Yes	No/Yes
ICU admission	No	No	Yes	No	Yes
First-line therapy/ Improvement ^c^	IVIG/No; IVMP+ tocilizumab/yes	IVMP+ tocilizumab/yes	IVMP/yes	First: IVIG/yes; Second: IVMP/yes	IVMP/yes
Long-term therapy	Steroid taper; tocilizumab	Steroid taper; tocilizumab	Steroid taper	Steroid taper	Steroid taper
Follow-up Serum MOG-IgG titer	Neg (3 months)	1:10 (6 months) Neg (1 year)	Neg (3 months)	1:10 (3 months) Neg (6 months)	Neg (6 months)
mRS scores					
Peak of attack	2	3	4	4	4
At discharge	0	1	2	2	2
Last follow-up	0	0	0	0	1

^a^ First available documented data, unless otherwise noted.

^b^ EBV DNA reads were tested via mNGS.

^c^ Improvement is defined by resolution of clinical symptoms and improved CSF parameters.

^d^ Retrospective test results.

^e^ Regarding vaccination history, all patients received only routine immunizations as required by the national immunization program (e.g., hepatitis B vaccine, BCG vaccine).

CSF, cerebrospinal fluid; EBV, Epstein–Barr virus; IVIG, intravenous immunoglobulin; IVMP, intravenous methylprednisolone; WBC, white blood cell, Neg, negative, mRS, modified Rankin Scale.

Regarding treatment response, only three patients exhibited transient mild improvement in CSF parameters and symptoms after initial antiviral therapy, whereas all patients achieved substantial clinical improvement following corticosteroid administration. Refractory cases required additional immunotherapy, including intravenous immunoglobulin (IVIG) or the interleukin (IL)-6 receptor antagonist tocilizumab, which was well-tolerated and effective for relapse prevention. The favorable response to immunotherapy further supports the diagnosis of MOGAD. Notably, B cell depletion therapies such as rituximab appear less effective in MOGAD than in NMOSD (6). Accordingly, we administered tocilizumab to our refractory MOGAD patients (7), with favorable responses and durable relapse prevention. Ongoing clinical trials are evaluating IL-6 receptor antagonists, including satralizumab (NCT05271409) and tocilizumab (NCT06452537), for MOGAD treatment.

Although mNGS is a powerful tool for unbiased pathogen identification, EBV detection in CSF requires cautious interpretation. Cerebrospinal fluid, characterized by low cellularity and minimal nucleic acid background, offers a high signal-to-noise ratio for mNGS analysis (8). Furthermore, the mNGS assay used in this study implemented rigorous workflows for signal-to-noise discrimination, background sequence subtraction, and strict contamination control measures, with internal negative controls included in each batch (9). Accordingly, only sequences that remained positive after the exclusion of known reagent and environmental background microbes were considered for clinical interpretation. Therefore, the detection of low-abundance viral sequences in CSF should not be disregarded, as such findings may correlate with relevant clinical manifestations in some patients under specific clinical contexts (10–12). Likewise, negative mNGS findings should not be overlooked in patients with relevant clinical manifestations and serological evidence of EBV antibodies, as the interval between symptom onset and testing may extend beyond the optimal window of peak viral shedding. Therefore, the significance of EBV detection should be interpreted within the specific clinical context.

Importantly, alternative explanations for the observed co-occurrence of EBV-related findings and MOGAD should be considered. First, given the high global prevalence of EBV seropositivity, isolated EBNA- and VCA-IgG seropositivity may reflect incidental past infection rather than a pathogenic trigger (13). Second, low-level EBV DNA in the CSF may be nonspecific, potentially representing an immune footprint of prior infection (13). Third, MOG-IgG positivity may be an epiphenomenon of inflammatory CNS disorders (14). Fourth, autoimmune activation might be driven by para-infectious mechanisms associated with influenza and herpes simplex virus, not specifically with EBV (15, 16). Additionally, MOGAD attacks can be triggered by antecedent infections of unclear etiology (17).

To further investigate the proportion of MOGAD cases with concurrent EBV detection in CSF, we retrospectively reviewed a cohort of patients admitted to our hospital between December 2024 and March 2026. Patients were included if they presented with clinical manifestations including fever, headache (with or without impaired consciousness or seizures) and positive EBV DNA sequences identified by CSF mNGS (**Supplementary Table 1**). Among the total 31 enrolled patients, 6 were seropositive for MOG-IgG, 10 were seronegative for demyelinating antibodies, 9 had not undergone antibody testing, and the remaining 6 had other CNS autoantibodies (3 positive for GFAP-IgG and 3 positive for AQP4-IgG). Our findings suggest that the potential association between EBV presence and MOGAD requires further validation in a larger, prospective patient cohort.

EBV is a ubiquitous human herpesvirus known to modulate autoimmune responses through latent infection and periodic reactivation (4). Previous studies reported elevated antibody titers against the EBNA1_AA386–405_ epitope in patients with relapsing remitting MS or MOGAD, but not in those with neuromyelitis optica spectrum disorder (NMOSD) (18). Another investigation also documented increased EBNA1 IgG levels in MOGAD patients, although these were comparatively lower than those in MS patients (19). To date, only a few case reports have suggested a possible link between EBV infection and MOGAD. In 2017, Nakamura et al. described a patient who developed MOG IgG positive acute disseminated encephalomyelitis (ADEM) eight days after EBV related infectious mononucleosis (20). In 2022, Lin et al. reported a case of MOG IgG associated encephalitis with CSF restricted MOG IgG positivity, concurrent EBV infection, and Alzheimer’s disease like CSF changes (21). In 2025, Chen et al. presented two cases of EBV-associated MOGAD that improved with combined methylprednisolone and ofatumumab (22). Together with these observations, the potential clinical relevance of CSF EBV detection in our patients deserves careful consideration. Four patients presented with initial encephalitis-like manifestations at the time EBV was identified by CSF mNGS, and retrospective testing confirmed low MOG-IgG titers were already present during this period, suggesting a temporal correlation between EBV detection and disease onset.

This study has several limitations. First, it is a retrospective case series with a small sample size, and we did not pre-define a clear patient selection strategy, which inherently introduces referral and selection bias. Second, the study lacked a control group, which restricts us from ruling out the possibility that EBV reactivation is a coincidental finding rather than a disease-related event. Third, evidence for EBV relies mainly on serology and mNGS, without quantitative PCR or lytic phase viral markers, limiting our ability to confirm active CNS infection versus systemic or bystander reactivation. Additionally, follow-up data on serial changes in EBV viral load and MOG-IgG titers were limited. We also recognize the challenges in interpreting low-titer EBV sequences detected by mNGS. Thus, a definite causal relationship between EBV and MOGAD needs to be validated in further studies.

In summary, this case series identifies a potential co-occurrence between MOGAD presenting as encephalitis and concurrent EBV related laboratory findings, suggesting that EBV-associated immune activation may coexist with the autoimmune process in MOGAD patients. Future large scale prospective cohort studies are required to validate this association, and explore underlying mechanistic links, which may help optimize diagnosis and treatment strategies for MOGAD patients. .

## Data Availability

The original contributions presented in the study are included in the article/**Supplementary Material**. Further inquiries can be directed to the corresponding authors.
