# Supplementary material for: Myelin oligodendrocyte glycoprotein antibody-associated disease presenting as encephalitis and concurrent Epstein-Barr virus detection: a case series
Source: Front Immunol. 2026 May 8;17:1788238. doi: 10.3389/fimmu.2026.1788238 (PMC13193921; doi:10.3389/fimmu.2026.1788238)
Supplement: Supplementary file 1 [file DataSheet1.pdf]

Supplementary Materials for

Myelin oligodendrocyte glycoprotein antibody-associated disease presenting as encephalitis and concurrent Epstein-Barr virus detection: a case series

Patient 6

A 33-year-old male admitted with a two-week history of stabbing headache, predominantly right-sided and worse during the daytime, accompanied by hand tremors. Physical examination revealed no abnormal neurological signs. CSF analysis showed elevated opening pressure (204 mmH_2O), increased protein (0.84 g/L), and leukocytosis (163 cells/mm^3) with 84% mononuclear cells. Serological testing revealed positive VCA-IgM, VCA-IgG and EBNA-IgG, with undetectable serum EBV DNA, while mNGS of CSF was positive for Epstein–Barr virus (3 reads). MOG-IgG was negative in serum. He was treated with penciclovir and discharged with substantial clinical improvement.

However, two weeks later, the patient was admitted due to fatigue, dizziness, and impaired concentration. Brain MRI showed swelling of the right cerebral cortex with linear, faint enhancement within the sulci. Serum MOG-IgG turned to be 1:32 (live CBA). Combined with these clinical, radiological, and serological findings, the diagnosis of MOGAD was established. He then received high-dose corticosteroid pulse therapy, after which his symptoms got relieved. Oral corticosteroids gradually tapered thereafter. Recent follow-up confirmed no clinical relapses.

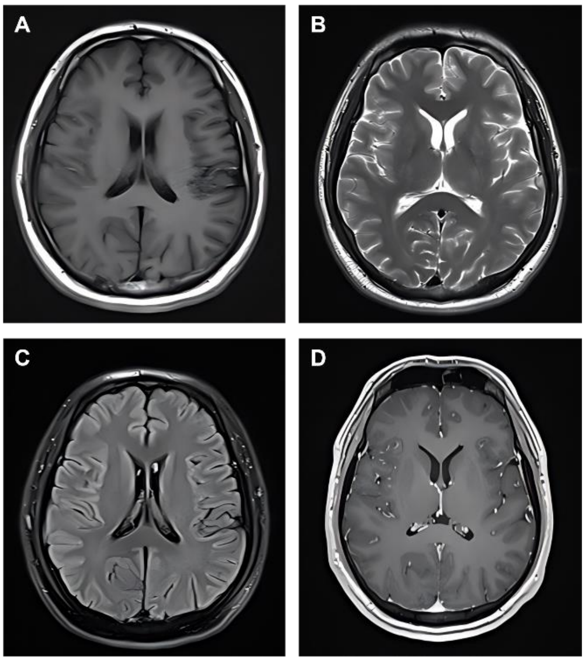

Figure S1. Imaging features of Patient 6.

Brain MRI revealed swelling of the right cerebral cortex, accompanied by linear faint enhancement within the sulci. A: T1-weighted image; B: T2-weighted image; C: T2-FLAIR image; D: Post-contrast image.

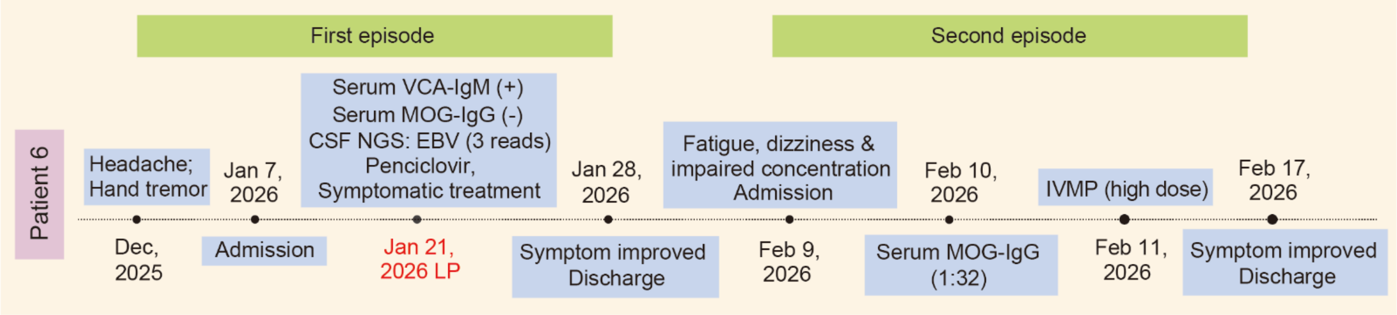

Figure S2. Clinical course of Patient 6.

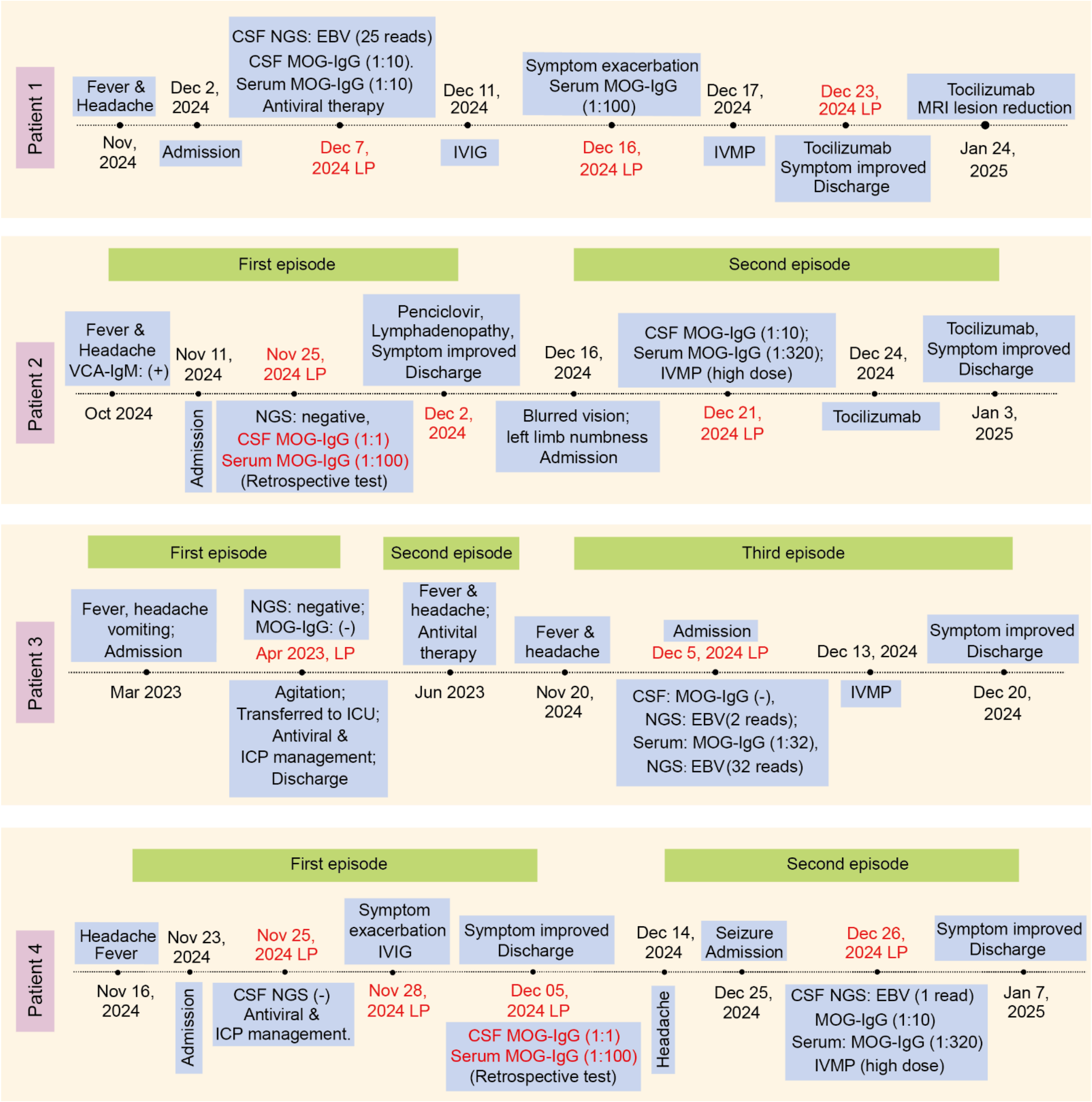

Figure S3. Clinical course of Patient 1–4 .

Table S1. Clinical characteristics of the EBV-positive patients (N=31) detected by mNGS in the CSF.

Item	MOG-IgG Pos	GFAP-IgG Pos	AQP4-IgG Pos	Ab Neg	Ab Untested
Number	6 (19.35%)	3 (9.68%)	3 (9.68%)	10 (32.26%)	9 (29.03%)
Age (median, min-max)	32.5 (24–46)	33 (21–70)	31 (48–59)	48.5 (36–73)	44 (36–71)
Male	5 (83.3%)	2 (66.7%)	1 (33.3%)	7 (70%)	7 (77.8%)
**Clinical manifestations**					
Headache	6 (100%)	1 (33.3%)	0	4 (40%)	5 (55.6%)
Fever	5 (83.3%)	3 (100%)	3 (100%)	6 (60%)	5 (55.6%)
Seizure	2 (33.3%)	2 (66.7%)	0	4 (40%)	2 (22.2%)
**Clinical subtypes**					
Meningitis	0	0	0	1 (10%)	2 (22.2%)
Encephalitis	6 (100%)	1 (33.3%)	0	3 (30%)	5 (55.6%)
Meningoencephalitis	0	0	0	2 (20%)	1 (11.1%)
Encephalomyelitis	0	1 (33.3%)	0	1 (10%)	1 (11.1%)
Meningoencephalomyelitis	0	0	0	1 (10%)	0
Myelitis	0	1 (33.3%)	2 (66.7%)	2 (20%)	0
Optic neuritis	0	0	2 (66.7%)	0	0
Area Postrema Syndrome	0	0	1 (33.3%)	0	0
**CSF abnormalities**					
Increased opening pressure	3 (50%)	2 (66.7%)	0	4 (40%)	7 (77.8%)
Leukocytosis	5 (83.3%)	3 (100%)	1 (33.3%)	10 (100%)	7 (77.8%)
Protein elevation	4 (66.7%)	3 (100%)	3 (100%)	7 (70%)	7 (77.8%)
**CSF NGS results**					
EBV mono-infection	6 (100%)	3 (100%)	3 (100%)	3 (30%)	5 (55.6%)
EBV + Bacteria	0	0	0	4 (40%)	1 (11.1%)
EBV + Fungi	0	0	0	0	1 (11.1%)
EBV + Herpesvirus	0	0	0	3 (30%)	1 (11.1%)
EBV + Other virus	0	0	0	0	1 (11.1%)
**Treatment**					
Antiviral	0	1 (33.3%)	0	0	4 (44.4%)
Antiviral + IVMP	3 (50%)	1 (33.3%)	0	7 (70%)	2 (22.2%)
Antiviral + IVMP + IVIG	1 (16.7%)	1 (33.3%)	0	3 (70%)	2 (22.2%)
Antiviral + IVIG + MAb	1 (16.7%)	0	0	0	0
Antiviral + IVMP + MAb	1 (16.7%)	0	2 (66.7%)	0	0
Antiviral + IVMP + IVIG + MAb	0	0	1 (33.3%)	0	0
Untreated	0	0	0	0	1 (11.1%)
**Death**	0	0	1 (33.3%)	0	1 (11.1%)

1.The monoclonal antibodies included in the table are inebilizumab, rituximab, tocilizumab, and eculizumab.

2. Abbreviations: Ab: Antibody; Pos: positive; Neg: negative; GFAP: Glial fibrillary acidic protein; MOG: Myelin oligodendrocyte glycoprotein; AQP4: Aquaporin-4; mNGS: metagenomic next-generation sequencing; ICP: intracranial pressure; IVMP: intravenous methylprednisolone; IVIG: intravenous immunoglobulin; MAb: monoclonal antibody.

Metagenomic next-generation sequencing (mNGS):

1. Nucleic acid extraction: DNA and RNA were extracted from CSF samples using the PathoXtract® Universal Nucleic Acid Extraction Kit (Magnetic Bead Method) (WYXM10002D-50), respectively.

2. Library Preparation: Extracted RNA was reverse-transcribed into complementary DNA (cDNA) using the PathoXtract® RNA Purification Kit (WYCM06601D-24). Purified DNA and synthesized cDNA were combined at equal concentrations, followed by genomic fragmentation, end repair, adapter ligation, and library amplification using the PathoLib™ Genome Fragmentation Kit (WYLM044032D-96) and PathoLib™ Genome Library Enrichment Kit (WYLM04407S-96). Batch-level nuclease-free water negative controls were included during library preparation to monitor potential reagent or environmental contamination.

3. High-throughpt sequencing: Qualified libraries were subjected to single-end 50-bp sequencing on the MGISEQ-200 platform. Approximately 10 million raw sequencing reads were generated per sample to ensure sufficient depth for pathogen detection, especially for low-abundance microorganisms in CSF.

4. Data Quality Control: Raw FASTQ-format data generated from sequencing is subjected to quality control and assessment using Trimmomatic v0.40. Low-quality or undetected sequences, adapter-contaminated sequences, highly redundant sequences with high coverage, and excessively short reads are filtered out to retain only high-quality sequencing data. Subsequently, the high-quality sequences are aligned against the human reference genome GRCh37 (hg19) using Bowtie2 v2.4.3 to remove human-derived sequences, thereby obtaining clean data suitable for subsequent pathogen identification. The resulting clean data are then compared against a pathogen database (NCBI GenBank/RefSeq/nt) using Kraken2 v2.1.0 to annotate species information of the pathogenic microorganisms, yielding the final microbial analysis and identification results. A curated background database of common reagent and environmental microbial contaminants was applied to filter false-positive signals derived from laboratory or kit-derived contamination.

5. Interpretation of Positive Results: The number of sequences for each microorganism is calculated using RPTM (reads per ten million, the number of microorganism-specific sequences detected per ten million sequencing reads)[1-2]. The threshold for viral positivity is RPTM⩾3. For bacteria and fungi, the RPTM threshold is RTPM⩾20. For special pathogens (Cryptococcus and Mycobacterium tuberculosis), a result is reported as positive when RPTM⩾1.

Final pathogenicity assignment was based not only on RPTM values but also on comprehensive evaluation of contamination risk, biological plausibility, and clinical correlation, including patient immune status, clinical manifestations, laboratory indices, and neuroimaging findings consistent with detected pathogen.

References:

1. Wilson MR et al. Clinical Metagenomic Sequencing for Diagnosis of Meningitis and Encephalitis. NEJM 2019;380:2327 – 2340；

2. Benoit, P., Brazer, N., de Lorenzi-Tognon, M. et al. Seven-year performance of a clinical metagenomic next-generation sequencing test for diagnosis of central nervous system infections. Nat Med 30, 3522 – 3533 (2024).
